# Frontal affinity chromatography to investigate the interaction of benzophenone with humic acid supported on microbore monolithic columns

**DOI:** 10.1016/j.heliyon.2025.e42390

**Published:** 2025-02-07

**Authors:** Fernando H. do Nascimento, Jorge C. Masini

**Affiliations:** Departamento de Química Fundamental, Instituto de Química, Universidade de São Paulo, Av. Prof. Lineu Prestes 748, São Paulo, 05508-000, SP, Brazil

**Keywords:** Frontal chromatography, Polymer monoliths, Affinity, Emerging pollutants, Benzophenone, Weak interaction

## Abstract

The principles of frontal affinity chromatography were used to determine the sorption constants and sorption capacities of benzophenone on immobilized humic acid. Poly(glycidyl-co-ethylene dimethacrylate) monoliths were constructed inside microbore capillaries (12 cm long × 1.016 mm internal diameter) and further aminated with ethylenediamine. The free amine groups coordinated Cu(II), which served as an intermediate ligand to immobilize about 27.2–28.7 mg of humic acid per gram of polymer skeleton (or 93 ± 4 μg per cm of column). The reversible nature of the interactions with Cu(II) allowed to leach and reload humic acid, thus suggesting that a single Cu(II) modified column may be further explored to immobilize humic acids from different sources using the concept of exchangeable chemistries on a stable monolithic platform. Frontal affinity chromatograms were obtained by injecting 1000 μL of benzophenone solutions of various concentrations (1.11–112 μmol L^−1^) at 25 °C and pH 7.00 ± 0.1. The concentration-dependent elution volume enabled the construction of sorption isotherms that were fitted to Langmuir and Freundlich equations and the linearized Scatchard plot. The binding of benzophenone to the humic substance was ruled by two classes of interaction sites with $K_{L}$ = (1.2 ± 0.2) × 10^6^ and (6.7 ± 0.8) × 10^3^ L mol^−1^ and a maximum sorption capacity of 19.2 ± 1.2 μmol g^−1^. The results correspond to an average of duplicate injections in three columns, thus demonstrating the acceptable reproducibility and stability of the proposed methodology.

## Highlights

• Frontal affinity chromatography on humic acid-supported stationary phase.
• Benzophenone retained by immobilized humic acid.
• Concentration-dependent retardation enables computing binding affinity and capacity.
• Cu(II) on monolithic support is a versatile platform to immobilize humic acid reversibly.

## Graphical abstract

**Figure:**
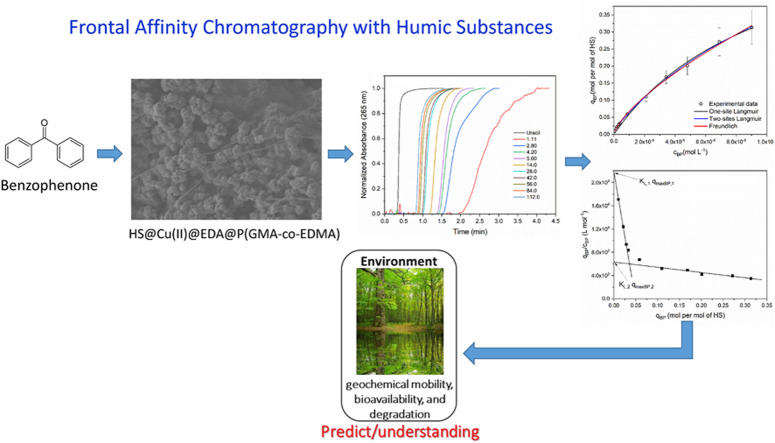

## 1 Introduction

Humic substances (HS) are major components of Natural Organic Matter (NOM) in fresh and seawaters, soils, and sediments. In natural freshwaters, they account for about 50 % of NOM. Hydrophobic moieties and polar groups such as carboxylic, phenolic, hydroxyl, and amines are some of the functional groups in the HS that hold together their supramolecular structures through hydrogen bonds. This chemical heterogeneity enables multi-mode interaction mechanisms of HS with natural and synthetic organic chemicals, playing a major role in their transport and bioavailability [[1], [2], [3], [4], [5], [6], [7], [8]].

Modeling the availability of emerging pollutants depends on the first step of determining their weak binding constants with HS. Fluorescence spectroscopy [9,10], UV–Vis spectroscopy [11], equilibrium dialysis [12,13], and solid-phase extraction combined with liquid chromatography-mass spectrometry [14,15], batch adsorption in 0.01 mol L^−1^ CaCl_2_ followed by HPLC analysis [16] have been employed to elucidate interactions of humic acids with organic micropollutants. These methods demand large sample volumes, long equilibration times, and laborious extraction steps which may imply analytical errors and experimental artifacts [[12], [13], [14],^17^].

Zonal and frontal high-performance affinity chromatography (HPAC) have been proposed as new and alternative methods to investigate the interactions of humic substances with herbicides, pharmaceuticals, and emerging pollutants. HPAC involves the immobilization of the HS in an inert support that enables ready accessibility of the studied pollutant to the HS functional groups. Besides, the stationary phase must be stable under changes in chromatographic parameters (pH, ionic strength, temperature, etc.), and suffer negligible non-specific interactions. Following these guidelines, Iftekhar et al. prepared a non-covalently entrapped humic acid stationary phase [18]. On the other hand, binding humic structures covalently to the support may be advantageous over the non-covalent entrapment to circumvent the potential limitations imposed by the supramolecular properties of humic acids [19].

Porous organic monoliths are suitable supports in HPAC because they can easily retain large molecules and supramolecular structures from proteins, humic acids, and viruses [20] at the pore surface exploring several mechanisms provided by the reactivity of the functional monomers. Glycidyl methacrylate (GMA) is a versatile monomer since various reagents can functionalize its epoxy group conferring ion exchange and affinity retention modes [[21], [22], [23], [24]]. Guillaume and Andre, for instance, immobilized biotinylated humic acid onto a neutravidin-grafted poly(glycidyl methacrylate-co-ethylene dimethacrylate), poly(GMA-co-EDMA), monolithic capillary (20 μm i. d.) to investigate the binding of rodenticides and severe acute respiratory syndrome coronavirus-2-spike proteins [25]. We explored the concepts of immobilized metal ion affinity and the strong affinity of humic acid to Cu(II) ions to prepare an HS-loaded poly(GMA-co-EDMA) monolithic columns that were first functionalized with ethylenediamine (EDA) to provide a chelating pore surface to hold Cu(II) that in turn retained HS also by surface complexation. This column enabled us to compare the affinity of caffeine, atrazine, propazine, simazine, and benzophenone with the immobilized HS using zonal chromatography [19].

Benzophenone (BP) is the simplest diaromatic ketone naturally occurring in fungi, plants, and fruits, acting as a protective agent, and shielding plants from radiation damage by absorbing UV light [26]. It has been widely used in sunscreen formulations so it has been detected worldwide in surface waters, groundwater, and sediments [27]. Currently, several studies have categorized benzophenone as a skin-allergenic [28], persistent, bio-accumulative, toxic, and a possible human carcinogen and endocrine disruptor [29,30]. Preliminary studies have demonstrated possible links between benzophenone exposure and mortality in developing coral, coral bleaching, and genetic damage to coral and marine life [31]. Thus, studies are needed to investigate the fate of benzophenone in environmental compartments [32]. In this study, we examined the interaction of benzophenone with HS-loaded poly(GMA-co-EDMA) monolithic columns using frontal chromatography. Unlike zonal chromatography, frontal chromatography allows the application of the Langmuir equation to estimate both the free energy-related binding constant and the binding capacity. Zonal chromatography utilizes small sample volumes and rapid analyses, whereas frontal chromatography involves the continuous pumping of the analyte or injecting a sample volume significantly exceeding the column volume. This process continues until equilibrium is reached, where the analyte concentration at the column end matches the input concentration. To address the long analysis time associated with this method, we propose using columns with microbore dimensions, thus avoiding the nanoscale for which specialized chromatographs are necessary.

## 2 Experimental

### 2.1 Apparatus and reagents

The monomers glycidyl methacrylate (GMA) and ethylene glycol dimethacrylate (EDMA) were purchased from Sigma-Aldrich (St. Louis, MO, USA) and passed through a basic aluminum oxide column to remove polymerization inhibitors. N-propanol and 1,4-butanediol were obtained from Sigma-Aldrich (used as received) and served as porogenic solvents. Azobisisobutyronitrile (AIBN) from Sigma-Aldrich was the free-radical initiator. HPLC-grade methanol was from Merck KGaA (Darmstadt, Germany). Ethylenediamine (EDA) was from Sigma-Aldrich. Aldrich sodium salt of humic acid (lot STBB1688) was used as the model of humic substances. A working humic acid solution was prepared by dissolving the sodium salt in deionized water, with subsequent pH adjustment to 7.0 ± 0.1 by adding 1.0 mol L^−1^ hydrochloric acid. This solution was filtered through 0.45 μm membranes and stored in a refrigerator. The concentration of soluble humic acid was determined by the dry weight of 5.00 mL aliquots, dried in a vacuum oven at 60 °C until constant weight, resulting in a concentration of 633.3 mg L^−1^. Deionized water (resistivity >18 MΩ cm) was obtained from a Simplicity 185 system from Millipore (Billerica, MA, USA). Fused-silica-lined stainless-steel (Silcosteel™) tubing with 1.59 mm o.d. and 1.016 mm i.d. was purchased from Restek Corporation (Centre County, PA, USA) and used to fabricate the semi-micro columns.

A 1000 mg L^−1^ stock solution of BP (Sigma-Aldrich) was prepared in HPLC-grade methanol and stored in a refrigerator. Working solutions within the concentration range between 0.20 and 20 mg L^−1^ BP (1.11–112 μmol L^−1^) were prepared in 2 % (v v^−1^) methanol in 10 mmol L^−1^ phosphate buffer solution (PBS), pH 7.00.

Chromatographic experiments were made in a Dionex Ultimate 3000 Dual Micro LC system (Dionex Softron GmbH, ThermoFisher Scientific, Germany) using dual micro DGP-3600 RS pumps with an SRD-3600 in-line degasser, a TCC 3000SD column compartment and an MWD-3000 UV/Vis detector coupled to a 2.5 μL semi-micro flow cell. For manual injection of larger sample volumes (500–2000 μL), a two-way, six-port 9010 Rheodyne injection valve was coupled to the chromatograph. The software Chromeleon® 6.8 controlled the instrument, data acquisition, and data processing. Connections of the column tube to the chromatographic system were made using P-742 PEEK ZDV unions for 1/16 inch o.d. from IDEX Health and Science (Oak Harbor, WA, USA).

### 2.2 Functionalization of the inner wall of the chromatographic tubes

The inner wall of the Silcosteel™ was sequentially washed with ethanol, water, 0.2 mol L^−1^ NaOH (60 min at 5 μL s^−1^), water, 0.2 mol L^−1^ HCl (60 min at 5 μL s^−1^), water and ethanol. Next, the tube was filled with 20 wt% 3-(trimethoxysilyl)propyl methacrylate in 95 % (v v^−1^) ethanol (apparent pH adjusted to 5.0), previously sonicated for 5 min. The ends of the tube were closed with pieces of Pharmed® peristaltic pump tubes sealed with solid PTFE tubes. The system was heated overnight at 60 °C inside the oven of a gas chromatograph. Finally, the tube was washed with acetone, dried in a flow of N_2,_ and cut into pieces of approximately 15 cm [33].

### 2.3 Preparation and modification of the poly(GMA-co-EDMA) monoliths

Three columns were prepared. A polymerization mixture containing 24 wt% GMA, 16 wt% EDMA, 45,5 wt% 1-propanol, and 14,5 wt% 1,4 butanediol was prepared in a 2 mL amber vial in the presence of 1.0 wt% AIBN (concerning the monomers) [34,35]. The mixture was sonicated (10 min), purged with N_2_ (10 min), and used to fill the activated Silcosteel™ tubes, which were closed on both ends, vertically positioned inside a gas chromatograph oven and heated at 60 °C for 24 h. Next, the columns were flushed with ACN at 500 μL min^−1^ using an HPLC pump until a constant pressure and then with water to remove ACN. The obtained poly(GMA-co-EDMA) columns were immersed in a water bath heated at 80 °C and flushed (8 μL min^−1^) with 10 mL of 50 % (v v^−1^) EDA in water using the infusion pump. After functionalization with EDA, the columns were washed with deionized water to remove the reagent excess [36].

To saturate the complexing sites of the EDA-modified poly(GMA-co-EDMA) with the Cu(II), 20 mmol L^−1^ CuSO_4_ (2.0 mL) was pumped through the columns at 250 μL min^−1^ using one of the micro DGP-3600 RS pumps coupled to a two-way, six-ports 9010 Rheodyne injection valve fitted out to a 2 mL loop. The column end was connected to a 1-cm optical path, 2.5 μL semi-micro flow cell of the MWD-3000 UV/Vis detector for spectrophotometric monitoring of the breakthrough curve at 790 nm. Loading of HS followed a similar procedure as used for Cu(II) but now measuring the absorbance at 500 nm.

The mass of loaded HS was determined by either the breakthrough curve or by extracting the immobilized HS with NaOH. This extraction was made with 2.0 mL of 0.10 mol L^−1^ NaOH at 100 μL min^−1^ for 20 min and the extracted solution was diluted to 6.0 mL with 0.10 mol L^−1^ NaOH. The concentration of extracted humate was determined spectrophotometrically at 500 nm by a calibration curve constructed with humic acid solutions (40–600 mg L^−1^) prepared in 0.10 mol L^−1^ NaOH.

The reproducibility of the columns was evaluated by the relative standard deviations (RSD) of permeabilities, breakthrough times of benzophenone, and amount of immobilized humic acid. Experiments were made in duplicate in the three synthesized columns.

### 2.4 Chromatographic experiments

The mobile phase used in this work was 10 mmol L^−1^ PBS pumped at 250 μL min^−1^ for at least 10 min to equilibrate the column. The column compartment was thermostated at 25 °C and the injection volume was 1000 μL. The injected solutions were at concentrations, $c_{BP}^{0}$, 1.11, 2.80, 4.20, 5.60, 13.99, 27.99, 41.98, 55.98, 83.97, and 112.0 μmol L^−1^, all in 2 % (v v^−1^) methanol and 10 mmol L^−1^ PBS. Detection was made at 265 nm and the dead volume of the system was determined by injecting 1000 μL of a 0.11 mg mL^−1^ uracil solution in 2 % methanol and 10 mmol L^−1^ PBS. The total run time was 6 min, which allowed us to determine the breakthrough curve and condition the column for the next injection.

### 2.5 Computation

The amount of immobilized Cu(II) or HS (q) was computed as:

(1) $$q=\frac{\left(t-t_{0}\right)Qc}{L}$$

Where t is the elution time of Cu(II) or HS and $t_{0}$ is the elution time of an unretained substance such as acetone or uracil and serves as the dead-volume marker. Both t and $t_{0}$ were measured at normalized absorbance AA0=0.5 where A and $A^{0}$ are the absorbances read at the column outlet and inlet, respectively [25,37]; Q is the flow rate (L min^−1^); c is the concentration (mol L^−1^ for Cu(II), and mg L^−1^ for HS); and L is column length in cm.

Computation of the binding parameters by frontal chromatography assumed the breakthrough curves as symmetrical. It involved the determination of t and $t_{0}$, where t is the elution time of the studied substance (BP in the present case) in the breakthrough curve generated by plotting the normalized UV absorbance (AA0) versus time. t is measured at AA0=0.5. From $t_{0}$ and t, the respective elution volumes ($V_{0\;}$ and V) of the unretained and studied substances are obtained by multiplying the elution times by the flow rate.

The Langmuir equation provided the binding parameters:

(2) $$q_{BP}=q_{\max,BP\;}\frac{c_{BP}\;K_{L}}{1+c_{BP}K_{L}}$$

where:

$q_{\max,BP}$ is the maximum capacity of the immobilized HS to bind BP;

$K_{L}$ is the Langmuir constant, related to the Gibbs free energy of sorption;

$c_{BP}$ is the free concentration of BP. In frontal chromatography, the injected volume is much larger than that of the column, and as the stationary phase reaches the dynamic equilibrium with the analyte (noticed as a plateau instead of a sharp peak), $c_{BP}$, at the plateau, equals the initial concentration ($c_{BP}^{0}$);

$q_{BP}$ is the sorbed amount of BP per mass of HS equilibrated in the column for a given initial total concentration of BP, $c_{BP}^{0}$; $q_{BP}$ is computed as qBP=[cBP0(V−V0)m], where *m* is the immobilized mass of HS in the column. According to Sigma-Aldrich, the molar mass of the studied humic acid can range from 2 kDa–500 kDa, with the bulk in the 20–50 kDa range. Based on this information, we converted the immobilized mass of HS assuming a mean 35 kDa M mass, as used by Ifthekar et al. [18].

Computation of $q_{\max,BP}$ and $K_{L}$ was made by nonlinear regression fitting using the Origin 2020 64-bit Academic software (OriginLab Corporation, Northampton, MA, USA) [38].

## 3 Results and discussion

### 3.1 Characterizations

Three HS-loaded columns were prepared and their mean permeability, computed by the Darcy equation, was (4.56 ± 0.59) × 10^−14^ m^2^, which enabled working at a flow rate of 250 μL min^−1^ at backpressure that did not exceed 15 MPa. This permeability was consistent with that of columns previously prepared by UV-mediated polymerization inside 2 mm i.d. Polypropylene ink-pen tubes [19].

**Fig. 1:**
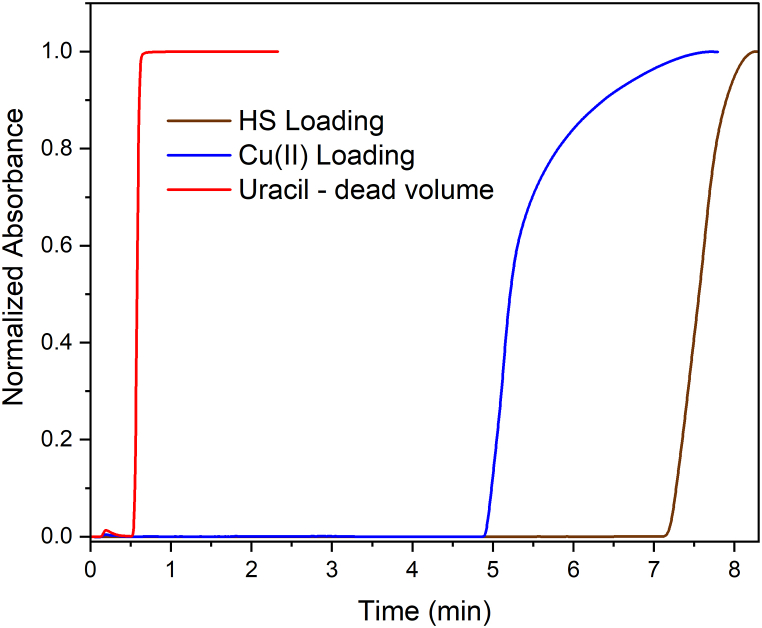
Normalized breakthrough curves for the determination of the dead volume of the EDA@poly(GMA-co-EDMA) column with 10 mg L^−1^ uracil (265 nm), followed by 20 mmol L^−1^ CuSO_4_ loading (790 nm) and then 633.3 mg L^−1^ humic substance (HS, pH 7.0, 500 nm). In all experiments, the column oven was thermostated at 25 °C, and the flow rate was set at 250 μL min^−1^. The carrier solution was deionized water and the injected volume of uracil, Cu(II), and HS was 2.0 mL.

For 12 cm long capillaries with 1.016 mm i.d., the internal volume is 97.2 μL, but as the polymerization mixture contains 40 wt% in monomers, and assuming 100 % yield in the polymerization reaction, it is expected the formation of 38.90 μL of polymer, the rest composed majorly by macropores. Assuming the density of the monomers is about 1.0 g cm^−3^, the total mass of the polymer skeletons should not exceed 38.90 mg, that is 3.24 mg per cm of column. Since the GMA corresponds to 60 wt% of the polymer mass, there is a maximum of 13.7 μmol of epoxy groups per cm of the columns, part available at the polymer pore surface and part embedded in the polymer matrix. After converting the epoxy groups in amine functionalities, the columns retained 1.7 ± 0.3 μmol Cu(II) cm^−1^. The excess of epoxy over the Cu(II) can be explained by the embedded portion that is not available for functionalization reactions with EDA and Cu(II). Breakthrough curves of the EDA@poly(GMA-co-EDMA) column with uracil ($V_{0})$, Cu(II), and then with HS are shown in Fig. 1.

**Fig. 2:**
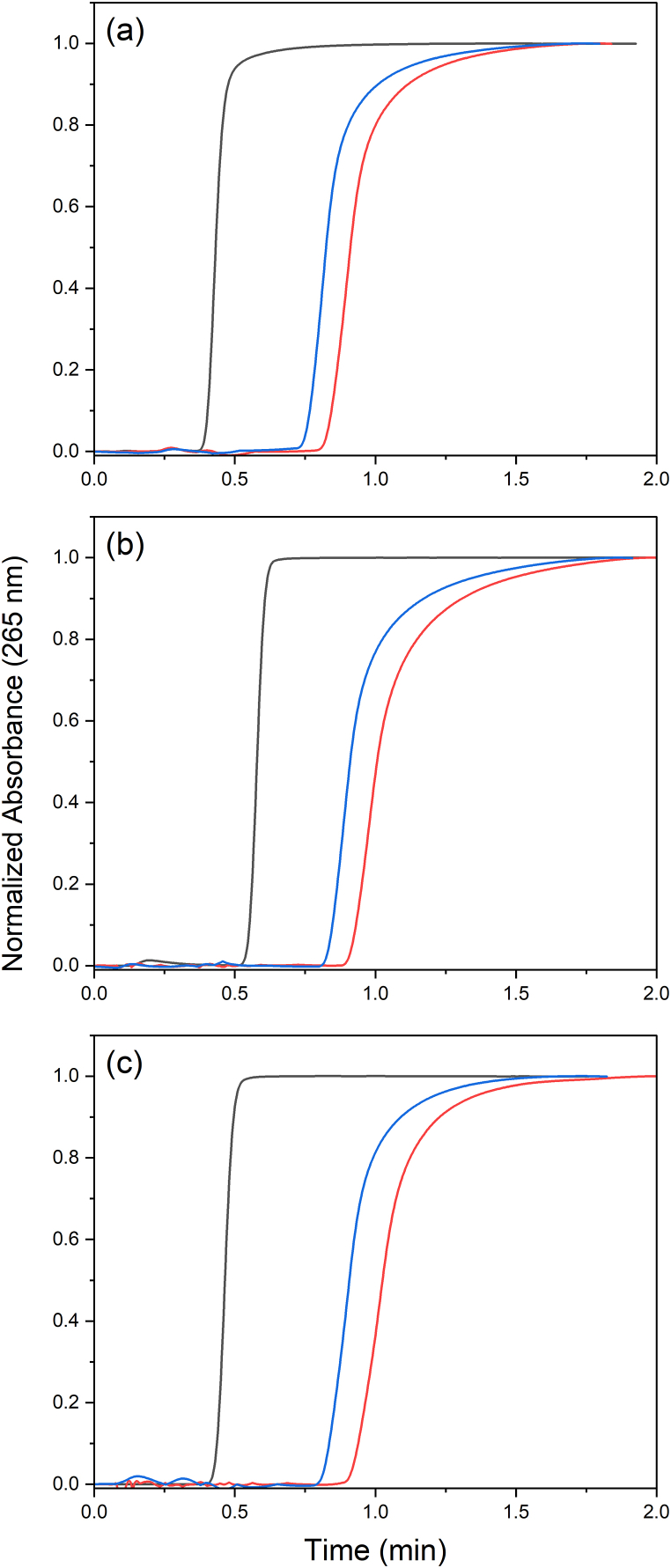
Frontal chromatograms of 10 mg L^−1^ uracil (black), 83.97 (red), and 112.0 (blue) μmol L^−1^ BP solutions in 10 mmol L^−1^ PBS (pH 7.0) at 25 °C in an HS@Cu(II)@EDA@poly(GMA-co-EDMA) column after (a) first HS loading, (b) second HS loading after first HS extraction, and (c) third HS loading after second HS extraction. Flow rate = 250 μL min^−1^. Injection volume = 1000 μL. Mobile phase: 10 mmol L^−1^ PBS (pH 7.0).

Using equation (1) and the breakthrough curves (Fig. 1) it is possible to estimate the immobilization of 0.0881 mg HS cm^−1^. Alternatively to the breakthrough curves, the amount of HS immobilized in the columns was determined by molecular spectrophotometry in 0.10 mol L^−1^ NaOH extracts. This alkaline treatment disrupts the hydrophobic and H-bonds that stabilize the supramolecular structures, releasing Na^+^-humates. The amount of released humate was 0.093 ± 0.004 mg cm^−1^. Thus, the mass of immobilized HS corresponded to about 2.7–2.9 % of the polymer mass (38.9 mg per 12-cm column). Additionally, the mass of HS determined by the breakthrough curve and NaOH extraction differed by only 5.3 % indicating that both approaches lead to consistent results. For one of the columns, the procedure of NaOH extraction followed by HS reloading was repeated twice and provided reasonably reproducible results in the frontal chromatography of BP. The $t_{0}$ values varied randomly by 15.7 % from the first to the third HS load, whereas the $\Delta_{t}$ varied 12.8 and 14.3 % for the 83.97, and 112.0 μmol L^−1^ BP, respectively (Fig. 2). An interesting consequence is that this column composition can be used to explore the concept of exchangeable chemistries [39] to investigate the behavior of HS from different sources without the need to prepare new columns for each sample. The treatment with NaOH released only the HS since no evidence of Cu(II) leaching was observed in qualitative tests with NH_4_OH. To break down the Cu(II) binding to EDA, a 1 mol L^−1^ HNO_3_ is necessary as formerly described in an SPE method to concentrate Cu(II) from reservoir waters [36]. This feature may be a versatile alternative to the strategies used by Iftekhar et al. [15], who entrapped the HS, and by Guillaume and André [25], who immobilized biotinylated humic acid onto a neutravidin-grafted poly(GMA-co-EDMA) column. In these later cases, each isolated HS sample requires the preparation of new column supports.

The amount of HS immobilized in the microbore monoliths synthesized by thermal initiation (38.9 mg HS per gram of polymer) was lower than that obtained by UV-induced polymerization inside 2 mm i.d. Polypropylene tubes (86.5 ± 0.8 mg g^−1^), suggesting the short polymerization times provided by UV-polymerization enhances the availability of functionalities able to immobilize EDA, and thus Cu(II) and HS. The amount of immobilized HS in the present work is consistent with that described by the entrapment method used by Ifthekar et al. (30–37 mg) [18]. The lower amount of immobilized humic acid was not an issue for the frontal affinity chromatography provided the benzophenone does interact with the monolithic skeleton as the results in section 3.2 demonstrate.

### 3.2 Control experiments

**Fig. 3:**
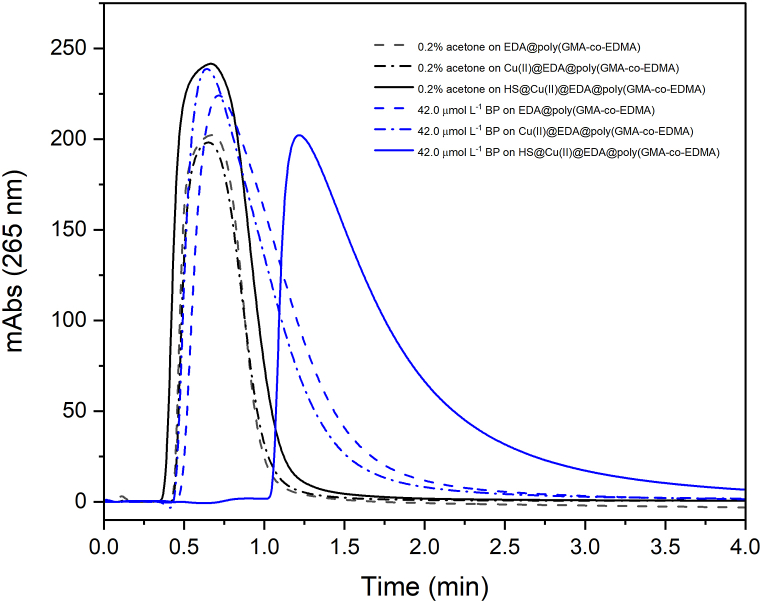
Control chromatograms in the monolithic poly(GMA-co-EDMA) columns that were modified with EDA, then Cu(II), and then HS. Flow rate = 250 μL min^−1^; BP solutions in 10 mmol L^−1^ PBS (pH 7.0); Temperature = 25 °C, Sample injection = 100 μL.

Injections of 112 μmol L^−1^ BP and 0.2 % (v/v) acetone (used as a dead volume marker) were made into columns modified with EDA and subsequently with Cu(II) (Fig. 3). The results showed no significant interaction, as the retention times of BP in these columns did not differ significantly from those of acetone. The lack of BP retention on the Cu(II)-loaded column indicates that cation-π stacking interactions between BP and Cu(II) can be ruled out, thus demonstrating that the retardation of BP on the HS-modified column is solely due to interactions with HS.

In contrast, cation-π interactions may play a significant role in the retention of HS on the Cu(II)-loaded column, as HS is rich in phenolic functionalities. Recent studies have shown that Cu(II) binds weakly to tryptophan via cation-π interactions [40]. Consequently, some phenolic groups in HS may not be available for interaction with BP through π-π mechanisms, which could be a limitation of the proposed strategy.

Ongoing work is evaluating the retention of HS through purely electrostatic attraction to protonated EDA. In a previous study, this electrostatic strategy retained much less HS, and the binding was less stable compared to coordination involving Cu(II) [19].

### 3.3 Column-to-column reproducibility

Besides the permeabilities, Cu(II), and HS loads, the three columns' reproducibility was evaluated by obtaining breakthrough curves at the concentration levels of benzophenone (1.0, 5.0, and 10.0 mg L^−1^). Thus, the breakthrough times (discounted the time of unretained substance) were 1.43 ± 0.12, 1.06 ± 0.07, and 0.93 ± 0.06 min, with RSD values of 9.0, 6.9 and 6.0 % for the 1.0, 5.0 and 10.0 mg L^−1^ BP, respectively. This column-to-column reproducibility is consistent with the poly(GMA-co-EDMA) columns prepared by photopolymerization, which, after modification with EDA, Cu(II), and HS exhibited a 6.8 % RSD of retention factor of BP (10 mg L^−1^) in zonal chromatography. A similar magnitude of RSD in the column reproducibility was described by Iftekhar et al. [18] with the entrapped HS-packed column retaining antibiotics. Guillaume and André [25] achieved better reproducibility (RSD >3 % in several conditions of pH and ionic strength) with their biotinylated humic acid immobilized onto poly(GMA-co-EDMA) monoliths prepared inside 20 μm i.d. Nano capillaries for the breakthrough times of rodenticides and SARS-CoV-2S protein.

The repeatability of the breakthrough time for a sequence of injections (n = 3 to 5) exhibited RSD <1.6 % for any column and or concentration, denoting excellent precision for the main chromatographic parameter used in frontal chromatography.

### 3.4 Frontal chromatography and sorption isotherms

**Fig. 4:**
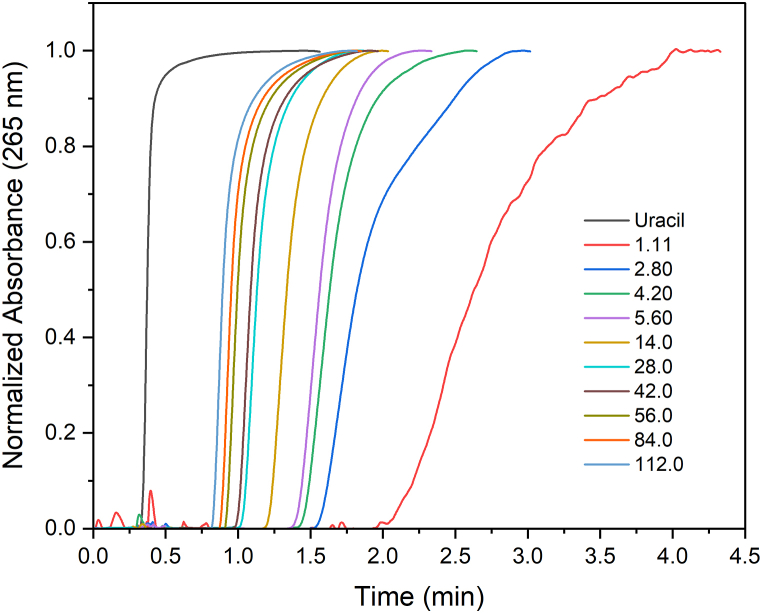
Typical frontal chromatograms of BP solutions with total concentrations ($c_{BP}^{0}$) varying from 1.11 to 112 μmol L^−1^ in 10 mmol L^−1^ PBS (pH 7.0) at 25 °C using an HS@Cu(II)@EDA@poly(GMA-co-EDMA) monolithic column (120 × 1.016 mm i.d.). Flow rate = 250 μL min^−1^. Injection volume = 1000 μL. Mobile phase: 10 mmol L^−1^ PBS (pH 7.0).

The main premise of frontal chromatography is that by continuously pumping BP through the affinity column, a dynamic equilibrium sets and the analyte concentration at the column exit ($c_{BP}$) equals that pumped through the column ($c_{BP}^{0}$). In the present work, the injected sample volume (1000 μL) was about 20-fold larger than the estimated internal empty column volume so $c_{BP}$ at the peak plateau equaled $c_{BP}^{0}$. At the plateau, the normalized absorbance (AA0 or cBPcBP0) corresponded to that of the dynamic equilibrium between BP and the immobilized HS (Fig. 4).

**Fig. 5:**
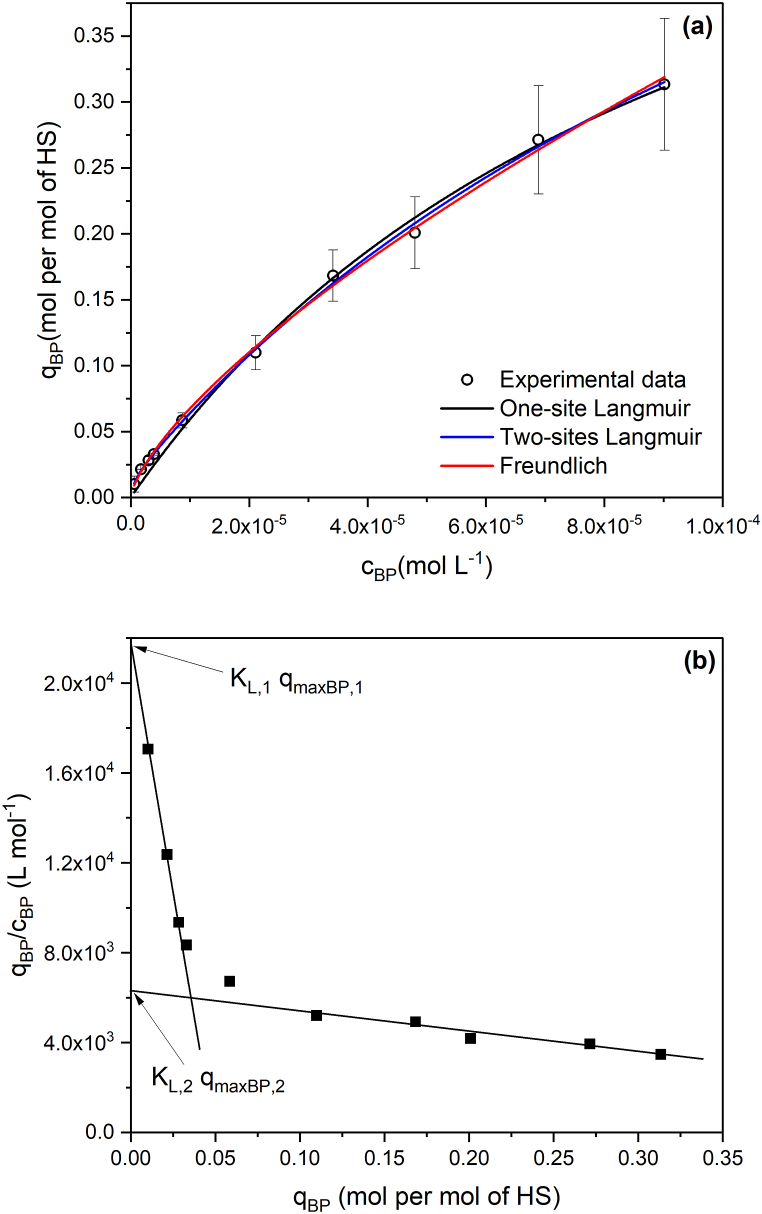
Sorption isotherm (a) of BP onto the immobilized HS fitted by nonlinear regression analysis using one-site and two-site Langmuir, and Freundlich models, and (b) Langmuir model linearized by the Scatchard equation.

The concentration-dependent retardation observed in Fig. 4 is a consequence of the competition among the BP molecules for the HS interaction sites. The elution times, and thus their respective elution volumes, decrease as the saturation level of the HS increases. The analysis of this phenomenon by the Langmuir equation (eq. (2)) provides the sorption constant ($K_{L}$) and the maximum sorption capacity ($q_{\max,BP\;}$) by nonlinear regression analysis (Fig. 5a) or graphically by linearizing equation (2). A typical linearized form is the Scatchard equation:

(3) $$\frac{q_{BP}}{c_{BP}}=-K_{L}q_{BP}+K_{L}q_{\max,BP}$$

From equation (3), the plot of qBPcBP versus $q_{BP}$ results in a straight line with ${-K}_{L}$ as the slope, which enables the computation of $q_{\max,BP}$ from the intercept. This assumption, however, is valid for homogeneous ligands, which is not the case for HS. In the present work, the Scatchard plot did not result in a single straight line but in a curvature that could be approached to two straight portions, thus suggesting the existence of interaction sites with distinct sorption energies (Fig. 5b) toward BP. The choice of points to make a linear fitting may be quite subjective [38], so we also fitted the experimental data by nonlinear regression analysis including a two-site Langmuir model (eq (4)) and the empirical Freundlich model (eq (5)) that is more suited to fit data coming from sorption on heterogeneous surfaces.

(4) $$q_{BP}=q_{\max,BP,1\;}\frac{c_{BP}\;K_{L,1}}{1+c_{BP}K_{L,1}}+q_{\max,BP,2\;}\frac{c_{BP}\;K_{L,2}}{1+c_{BP}K_{L,2}}$$

where indexes 1 and 2 refer to the interactions with larger and lower energies, respectively.

(5) $$q_{BP}=K_{F}c_{BP}^{1/n}$$

Where $K_{F}$ is the Freundlich constant and 1/n is the heterogeneity parameter which varies from 0 to 1. The closer the 1/n value is to the unity, the more homogeneous the adsorbent is, so the $K_{F}$ approaches a Henry-like constant. The fitted parameters were $K_{F}=\left(228\;\pm 4\right)$ 1/(mol L^−1^)^1/n^ and 1/n = 0.70 ± 0.02, with R^2^ > 0.995.

Table 1 shows the Langmuir sorption parameters found by linearization and nonlinear regression methods. The three Langmuir models yielded *q*_max_ values very close to 19 μmol g^−1^. The correlation coefficient was not very helpful in identifying the best model, as it was >0.94 in all cases. However, the relative standard deviation (RSD) of *q*_max_ adjusted by nonlinear regression for the one-site Langmuir model was quite high (31.5 %). For the two-site model, the RSD decreased to 7.8 %, indicating better suitability of this model for describing the experimental data. Linearization using the Scatchard method unequivocally indicated the presence of two adsorption sites and provided the *q*_max_ with the lowest RSD, just 0.52 %. Despite the differences observed by comparing the results obtained by linearization and nonlinear regression, especially for site 1, the constants are of similar magnitude and consistent with expected values for weak interactions, which are the interactions typically examined by frontal affinity chromatography [37]. The presence of stronger interaction sites of low abundance and more abundant weaker sites is a typical feature of humic substances [41,42]. This heterogeneity of binding interactions was observed by Cao et al. studying the interaction of BP with Amherst humic acid by deuterium exchanged and carbonyl ^13^C-labeled BP and ^1^H-^13^C NMR spectroscopy, finding that the affinity of BP for aromatic rings (phenolic moieties) by π-π stacking is greater than that interaction with carbohydrate and nonpolar alkyl components [43]. The Freundlich parameters obtained by Cao et al. were ${logK}_{F}=\left(2.69\pm 0.01\right)$ mg kg^−1^/(mg L^−1^)^1/n^ and 1/n = 0.87 ± 0.01 [43], suggesting that the interaction between Amherst humic acid and BP was more homogeneous than observed in our experiments with the Aldrich HS. The $\log\;K_{F}$ was of the same magnitude since converting for the same units, the value found in our experiments was ${logK}_{F}=\left(2.35\pm 0.04\right)$.

**Table 1:** Fitted Langmuir parameters obtained by linearization (Scatchard) and nonlinear regression analysis using one-site and two sites Langmuir equations (equations (2), (4)), respectively).

Model	$K_{L,1}$ (L mol^−1^)	$q_{\max,1}$ (μmol g^−1^)	R^2^	$K_{L,2}$ (L mol^−1^)	$q_{\max,2}$ (μmol g^−1^)	R^2^
Scatchard	(3.9 ± 0.2) × 10^5^	1.53 ± 0.05	0.94	(8._5_±1._2_) × 10^3^	19.2 ± 0.1	0.992
Two-site Langmuir^a^	(1.2 ± 0.2) × 10^6^	0.36 ± 0.08	–	(6.7 ± 0.8) × 10	19._2_ ± 1._5_	0.998
One-site Langmuir^a^	(10._1_±1._9_) × 10^3^	19 ± 6	0.995	–	–	–

a Obtained by Nonlinear Regression Analysis.

The chromatographic retention factor (*k*) of an injected substance should be directly proportional to a global affinity constant of the studied substance with the binding agent [18]. In our previous work, we determined a global affinity of (1.9 ± 0.3) × 10^3^ L mol^−1^ for the interaction between BP and the Aldrich HS using zonal affinity chromatography [19]. This affinity constant is of the same magnitude as that of *K*_*L,2*_ indicating the binding constants are in agreement with each other, independent of the analytical methodology. Benzophenone at lower concentrations binds preferentially to high affinity sites (high *K*_*L*_, low *q*) and is retained longer. As the concentration increases, BP molecules quickly saturate the high-affinity sites and start occupying the weaker sites, whose retention capacity is significantly lower, thus resulting in a reduced average retention time or a smaller retention volume, highlighting the heterogeneity of the ligand. The advantage of Frontal Affinity Chromatography is that the concentration-dependent retardation allowed us to investigate the affinity spectra that revealed the influence of two interaction sites, which was not possible by zonal chromatography since a single concentration was studied so the sites with a higher affinity were not detected.

## 4 Conclusion

This paper demonstrates that the porous poly(GMA-co-EDMA) polymer monolith can be efficiently modified with EDA to chelate Cu(II), which acts as an intermediate ligand between the surface pores and HS. The columns prepared in microbore capillaries (12 cm long × 1.016 mm i.d.) were permeable, stable, and reproducible, enabling the determination of binding parameters for the interaction between BP and the immobilized HS exploring the concept of frontal affinity chromatography. Therefore, they are a potentially useful platform for environmental research on the interactions between emerging pollutants and natural organic matter components. The results demonstrated that BP interacts with HS through at least two classes of interaction sites for which the Langmuir binding parameters (*K*_*L*_ and *q*_max_) were determined. The Freundlich parameters agreed with those reported for Amherst humic acid using deuterium exchanged and carbonyl ^13^C-labeled BP and ^1^H-^13^C NMR spectroscopy, validating the results of our simpler and cheaper methodology. Future work will explore the proposed monolithic platform to investigate the interaction of emerging pollutants and humic and fulvic acids isolated from other natural sources such as vermicompost.

## CRediT authorship contribution statement

**Fernando H. do Nascimento:** Writing – original draft, Methodology, Investigation, Formal analysis. **Jorge C. Masini:** Writing – review & editing, Supervision, Project administration, Funding acquisition, Formal analysis, Data curation, Conceptualization.

## Data availability statement

Data will be made available on request.

## Ethics declarations

The experiments did not involve humans or animals.

## Declaration of competing interest

The authors declare that they have no known competing financial interests or personal relationships that could have appeared to influence the work reported in this paper.

## References

[1] Angelico R., Colombo C., Di Iorio E., Brtnický M., Fojt J., Conte P. (2023). Humic substances: from supramolecular aggregation to fractal conformation—is there time for a new paradigm?. Appl. Sci..

[2] Chianese S., Fenti A., Iovino P., Musmarra D., Salvestrini S. (2020). Sorption of organic pollutants by humic acids: a review. Molecules.

[3] Lipczynska-Kochany E. (2018). Humic substances, their microbial interactions and effects on biological transformations of organic pollutants in water and soil: a review. Chemosphere.

[4] Piccolo A., Conte P., Cozzolino A. (2001). Properties of dissolved humic substances. Soil Sci..

[5] Piccolo A., Spark Donald S. (2002). Adv. Agron.

[6] Piccolo A., Conte P., Spaccini R., Chiarella M. (2003). Effects of some dicarboxylic acids on the association of dissolved humic substances. Biol. Fertil. Soils.

[7] Peng X.X., Gai S., Cheng K., Yang F. (2022). Roles of humic substances redox activity on environmental remediation. J. Hazard Mater..

[8] Hu B., Wang P., Wang C., Bao T. (2022). Photogeochemistry of particulate organic matter in aquatic systems: a review. Sci. Total Environ..

[9] Ferrie R.P., Hewitt G.E., Anderson B.D. (2017). A fluorescence quenching analysis of the binding of fluoroquinolones to humic acid. Appl. Spectrosc..

[10] Bai Y., Wu F., Liu C., Guo J., Fu P., Li W., Xing B. (2008). Interaction between carbamazepine and humic substances: a fluorescence spectroscopy study. Environ. Toxicol. Chem..

[11] Yuan X., Yang S., Fang J., Wang X., Ma H., Wang Z., Wang R., Zhao Y. (2018). Interaction mechanism between antibiotics and humic acid by UV-Vis spectrometry. Int. J. Environ. Res. Publ. Health.

[12] Sibley S.D., Pedersen J.A. (2008). Interaction of the macrolide antimicrobial clarithromycin with dissolved humic acid. Environ. Sci. Technol..

[13] Gu C., Karthikeyan K.G., Sibley S.D., Pedersen J.A. (2007). Complexation of the antibiotic tetracycline with humic acid. Chemosphere.

[14] Ding Y., Teppen B.J., Boyd S.A., Li H. (2013). Measurement of associations of pharmaceuticals with dissolved humic substances using solid phase extraction. Chemosphere.

[15] Alaghmand M., Alizadeh-Saei J., Barakat S. (2020). Adsorption and removal of a selected emerging contaminant, carbamazepine, using Humic acid, Humasorb and Montmorillonite. Equilibrium isotherms, kinetics and effect of the water matrix. J. Environ. Sci. Heal. - Part A Toxic/Hazardous Subst. Environ. Eng..

[16] Martínez-Mejía M.J., Sato I., Rath S. (2017). Sorption mechanism of enrofloxacin on humic acids extracted from Brazilian soils. Environ. Sci. Pollut. Res..

[17] Speltini A., Merlo F., Maraschi F., Sturini M., Contini M., Calisi N., Profumo A. (2018). Thermally condensed humic acids onto silica as SPE for effective enrichment of glucocorticoids from environmental waters followed by HPLC-HESI-MS/MS. J. Chromatogr., A.

[18] Iftekhar S., Poddar S., Rauhauser M., Snow D.D., Hage D.S. (2023). Preparation of entrapment-based microcolumns for analysis of drug-humic acid interactions by high-performance affinity chromatography. Anal. Chim. Acta.

[19] do Nascimento F.H., Masini J.C. (2024). Porous polymer monolithic columns to investigate the interaction of humic substances with herbicides and emerging pollutants by affinity chromatography. Anal. Chim. Acta.

[20] Vitek R., do Nascimento F.H., Masini J.C. (2021). Polymer monoliths for the concentration of viruses from environmental waters: a review. J. Separ. Sci..

[21] Svec F. (2010). Porous polymer monoliths: amazingly wide variety of techniques enabling their preparation. J. Chromatogr., A.

[22] Ribeiro L.F., Lopes Martins R., de Souza Costa D.M., Masini J.C. (2018). Poly glycidyl methacrylate-co-ethylene dimethacrylate porous monolith as a versatile platform for the development of separations and solid-phase extractions in sequential injection analyzers. J. Separ. Sci..

[23] do Nascimento F.H., Masini J.C. (2020). Immobilized metal affinity sequential injection chromatography for the separation of proteins. Anal. Lett..

[24] Vergara-Barberán M., Lerma-García M.J., Simó-Alfonso E.F., Herrero-Martínez J.M. (2023). Galactose-functionalized methacrylate polymers as affinity sorbents for extraction of food allergen lectins. Anal. Chim. Acta.

[25] Guillaume Y.C., André C. (2023). New liquid chromatography columns for highlighting the interaction of ligand candidates with humic acid. J. Separ. Sci..

[26] Marinov T., Kokanova-Nedialkova Z., Nedialkov P.T. (2023). Naturally occurring simple oxygenated benzophenones: structural diversity, distribution, and biological properties. Diversity.

[27] Gavrila A.A., Dasteridis I.S., Tzimas A.A., Chatzimitakos T.G., Stalikas C.D. (2023). Benzophenones in the environment: occurrence, fate and sample preparation in the analysis. Molecules.

[28] DiNardo J.C., Downs C.A. (2018). Dermatological and environmental toxicological impact of the sunscreen ingredient oxybenzone/benzophenone-3. J. Cosmet. Dermatol..

[29] Mustieles V., Balogh R.K., Axelstad M., Montazeri P., Márquez S., Vrijheid M., Draskau M.K., Taxvig C., Peinado F.M., Berman T., Frederiksen H., Fernández M.F., Marie Vinggaard A., Andersson A.M. (2023). Benzophenone-3: comprehensive review of the toxicological and human evidence with meta-analysis of human biomonitoring studies. Environ. Int..

[30] Matouskova K., Vandenberg L.N. (2022). Towards a paradigm shift in environmental health decision-making: a case study of oxybenzone. Environ. Heal. A Glob. Access Sci. Source..

[31] Cuccaro A., Freitas R., De Marchi L., Oliva M., Pretti C. (2022). UV-filters in marine environments: a review of research trends, meta-analysis, and ecotoxicological impacts of 4-methylbenzylidene-camphor and benzophenone-3 on marine invertebrate communities. Environ. Sci. Pollut. Res..

[32] Chaves M. de J.S., Barbosa S.C., de M. Malinowski M., Volpato D., Castro Í.B., dos S. Franco T.C.R., Primel E.G. (2020). Pharmaceuticals and personal care products in a Brazilian wetland of international importance: occurrence and environmental risk assessment. Sci. Total Environ..

[33] Masini J.C. (2016). Semi-micro reversed-phase liquid chromatography for the separation of alkyl benzenes and proteins exploiting methacrylate- and polystyrene-based monolithic columns. J. Separ. Sci..

[34] Henrique do Nascimento F., Trazzi C.R.L., Moraes A.H., Velasques C.M., Costa D.M. de S., Masini J.C. (2020). Construction of polymer monolithic columns in polypropylene ink-pen tubes for separation of proteins by cation-exchange chromatography. J. Separ. Sci..

[35] Peters E.C., Petro M., Svec F., Fréchet J.M.J. (1997). Molded rigid polymer monoliths as separation media for capillary electrochromatography. Anal. Chem..

[36] Ribeiro L.F., Masini J.C. (2018). Complexing porous polymer monoliths for online solid-phase extraction of metals in sequential injection analysis with electrochemical detection. Talanta.

[37] Kasai K. (2021). Frontal affinity chromatography: an excellent method of analyzing weak biomolecular interactions based on a unique principle. Biochim. Biophys. Acta Gen. Subj..

[38] Vitek R., Masini J.C. (2023). Nonlinear regression for treating adsorption isotherm data to characterize new sorbents: advantages over linearization demonstrated with simulated and experimental data. Heliyon.

[39] Cao Q., Xu Y., Liu F., Svec F., Fréchet J.M.J. (2010). Polymer monoliths with exchangeable chemistries: use of gold nanoparticles as intermediate ligands for capillary columns with varying surface functionalities. Anal. Chem..

[40] Yorita H., Otomo K., Hiramatsu H., Toyama A., Miura T., Takeuchi H. (2008). Evidence for the cation-π interaction between Cu2+ and tryptophan. J. Am. Chem. Soc..

[41] Zhang Z., Buffle J., Alemani D. (2007). Metal flux and dynamic speciation at (Bio)interfaces. Part II: evaluation and compilation of physicochemical parameters for complexes with particles and aggregates. Environ. Sci. Technol..

[42] Pinheiro J.P., Rotureau E., Duval J.F.L. (2021). Addressing the electrostatic component of protons binding to aquatic nanoparticles beyond the Non-Ideal Competitive Adsorption (NICA)-Donnan level: theory and application to analysis of proton titration data for humic matter. J. Colloid Interface Sci..

[43] Cao X., Lattao C., Pignatello J.J., Mao J., Schmidt-Rohr K. (2014). Sorption selectivity in natural organic matter probed with fully deuterium-exchanged and carbonyl-13C-labeled benzophenone and 1H-13C NMR spectroscopy. Environ. Sci. Technol..

